# Headache-attributed burden and a health-care needs assessment in Delhi and National Capital Region of India: estimates from a cross-sectional population-based study

**DOI:** 10.1186/s10194-025-02036-w

**Published:** 2025-05-07

**Authors:** Anand Krishnan, Debashish Chowdhury, Ashish Duggal, Ritvik Amarchand, Andreas Husøy, Timothy J. Steiner

**Affiliations:** 1 All India Institute of Medical Sciences, New Delhi, India; 2 GB Pant Institute of Postgraduate Medical Education and Research, New Delhi, India; 3 Department of Neuromedicine and Movement Science, NorHead, Norwegian University of Science and Technology (NTNU), Trondheim, Norway; 4 Department of Neurology, University of Copenhagen, Copenhagen, Denmark; 5 Division of Brain Sciences, Imperial College London, London, UK; 6 Department of Neuromedicine and Movement Science, Norwegian University of Science and Technology (NTNU), Edvard Griegs gate, Trondheim, Norway

**Keywords:** Headache, Migraine, Tension-type headache, Medication-overuse headache, Population-based study, Burden of disease, Health-care needs assessment, India, South East Asia region, Global Campaign against Headache

## Abstract

**Background:**

We have previously shown headache to be highly prevalent in Delhi and National Capital Region of northern India, as we did earlier in Karnataka State in the south. Here we present a complementary study performed contemporaneously of headache-attributed burden, along with a population health-care needs assessment.

**Methods:**

In a cross-sectional study using the standardised methodology of the Global Campaign against Headache, we randomly selected households, and one member aged 18–65 years from each, making unannounced visits. Trained interviewers used the HARDSHIP questionnaire incorporating enquiry into various aspects of headache-attributed burden: symptom burden, lost health, impaired participation in daily activities, quality of life (QoL) and willingness to pay (WTP) for treatment. Enquiry included questions about headache yesterday (HY).

**Results:**

Of *N* = 2,066, participants reporting headache in the past year spent 9.5% of their time with headache of moderate intensity (1.8 on the scale of 1–3). Population-level estimates of all time spent with headache were in the range 5.5–6.6%. On this measure, migraine (8.2%) was, at individual level, much more burdensome than tension-type headache (TTH) (1.7%), and females with migraine or TTH were more burdened (8.7% and 2.0% respectively) than males (6.0% and 1.0%). Migraine accounted for substantial health loss (3.6%) at individual level (disability weights from the Global Burden of Disease study factored in), but both measures of overall burden (QoL and WTP) found it greatest among those with probable medication-overuse headache (pMOH) or other causes of headache on ≥ 15 days/month (H15+), with TTH least. For all headache types, participation was more impaired in household than in paid work, the latter being little affected (overall, males 0.3 lost days/month, females 0.1). Impaired participation in social or leisure activities was close to unmeasurably low. Impaired participation from HY was 1.8% across all domains of activity. One quarter (26%) of the population aged 18–65 years would be expected to benefit from health care, meeting our criteria for need: 16.1% with migraine, 6.4% with H15+.

**Conclusion:**

Headache disorders in northern India are not only prevalent but also associated with high burden. One quarter of the adult population would benefit from professional headache care.

## Background

We have recently shown headache disorders to be highly prevalent among adults in Delhi and the National Capital Region (NCR) of India [1], with an age- and gender-adjusted 1-year prevalence of any headache of 67.9%. This finding in northern India was comparable with our earlier finding (62.0%) in a similar study in Karnataka State in the south of the country [2, 3]. In Karnataka we found commensurately high burdens associated with headache disorders, especially migraine [4]. We concluded then that, despite the existence of effective treatments, the limited access to health care was failing to alleviate headache-attributed symptom burden and impaired participation in daily activities [4].

Here we present not only headache-attributed burden in the adult general population of Delhi and NCR, with population-level estimates of time with headache and impaired participation, but also a population health-care needs assessment expressly to inform local and national makers of health policy and guide the allocation of limited health resources. Our estimates include lost productivity, to inform makers of economic policy also.

This paper continues the series of studies [2–13] using standardized methodology [14, 15] conducted within the Global Campaign against Headache in order to establish the scope and scale of the global burden of headache. The study was the third in South East Asia Region (SEAR), after the study in Karnataka [4] and another in Nepal [7].

## Methods

The methodology, fully documented previously [1, 16], is described here only in summary.

### Ethics

The study was conducted in accordance with the Declaration of Helsinki [17] and with approval from the Institutional Ethics Committee of Maulana Azad Medical College and Associated Hospitals, New Delhi.

All participants gave written consent before enrolment.

### Study design and sampling

This was a cross-sectional survey using a standardized sampling procedure and questionnaire [14, 15] carried out between December 2018 and June 2019. The design and sampling process have been described in detail before [16].

Multistage random sampling was used to generate a sample demographically, geographically and socioeconomically representative of the adult general population aged 18–65 years of the region. Randomly selected households, both urban and rural, were visited unannounced. One adult member (aged 18–65 years) of each, also randomly selected, was interviewed face-to-face by one of four interviewers. All were fluent in English and Hindi, had experience in the conduct of community-based surveys and were given one week of training in headache and the nature, methodology and purposes of the study.

According to guidelines [14], we planned for a sample of *N* > 2,000.

Interviews followed the structured Headache-Attributed Restriction, Disability, Social Handicap and Impaired Participation (HARDSHIP) questionnaire [15], either in the original English [15] or validated Hindi versions [16]. Modules of HARDSHIP covered various aspects of burden (reported below). The timeframes of enquiry were the preceding 1 year, 3 months, 1 month and 1 day, the last focused on headache yesterday (HY).

### Analyses

#### Headache diagnoses

These, according to the characteristics of the most bothersome headache only (when more than one headache type was reported), were based on ICHD-3 [18] and made algorithmically [15] during analysis. First, participants reporting headache on ≥ 15 days/month (H15+) were identified. They were classified as having probable medication-overuse headache (pMOH) when also reporting use of acute medication on ≥ 15 days/month or, otherwise, as having “other H15+” (which was not further diagnosed). In those remaining, definite migraine, definite tension-type headache (TTH), probable migraine and probable TTH were identified in this order according to the hierarchy of ICHD [18]. Definite and probable diagnoses were combined in further analyses.

#### Symptom burden

Usual headache intensity, reported subjectively as “not bad”, “quite bad” or “very bad”, was analysed by converting these responses onto a numerical scale 1–3 and treating as continuous data. Headache frequency was measured in days/month and usual headache duration in hours. The proportion of time in ictal state (pTIS) at individual level was calculated as the product, in hours, of headache frequency and duration (capped, for this analysis, at 24 h to avoid overestimation since frequency was in days/month) divided by total hours available (30*24).

Lost health was computed for migraine and TTH as the product of pTIS and the disability weight (DW) for each, on a scale 0–1, used by the Global Burden of Disease (GBD) study (migraine: 0.441; TTH: 0.037) [19].

#### Impaired participation

Impaired participation was evaluated through the Headache-Attributed Lost Time (HALT-30) index [20], which was a module within HARDSHIP [15], counting reportedly affected days in the preceding month. In the analysis, as recommended, “less than half achieved” was equated to “nothing achieved” and, in counterbalance, “more than half achieved” to “everything achieved” [20]. Impaired participation was assessed separately for paid (income-generating) work, household work (the essential chores of daily life) and social or leisure activities. Impaired participation in the first two of these domains constituted lost productivity.

#### Headache yesterday (HY)

In those reporting HY, headache duration and intensity and impaired participation yesterday were recorded. Impaired participation, in all activities as a whole, was assessed in the same manner as HALT data: dichotomized as either 0% (“more than half” or “everything” done) or 100% (“less than half” or “nothing” done).

#### Overall burden

Two measures were used to assess overall burden. Quality of life (QoL) was measured by WHOQoL-8, scoring responses to each of its 8 items on a scale 1–5 and summing these for a total in the range 8–40, higher scores indicating better QoL [21]. Willingness to pay (WTP) for effective treatment, derived by the bidding-game method [15], was reported in Indian rupees (INR) per month (on June 1st 2019, USD 1 = INR 65.58 [22]).

Both measures were considered to yield continuous data.

#### Health care needs assessment

To assess population need for headache-related health care, we counted all those we believed were likely to benefit from professional health care. We used the following criteria: (a) having H15+ (pMOH or other); (b) having migraine on ≥ 3 days/month; (c) having migraine or TTH *and* pTIS > 3.3% *and* moderate-to-severe headache intensity; (d) having migraine or TTH *and* losing ≥ 3 work and/or household days in the preceding 3 months (estimated from HALT data for 1 month multiplied by three).

#### Population-level estimates

These, for pTIS and impaired participation, were made from mean individual-level estimates, factoring in headache prevalence (1-year or 1-day) [1]. We made separate estimates from recalled and HY data.

All these estimates were adjusted for age and gender.

### Statistics

We used means with standard errors (SEMs), medians or proportions (%) with 95% confidence intervals (CIs), as appropriate.

Continuous variables were compared using ANOVA-tests and categorical variables using chi-squared tests.

Statistical analyses were executed using RStudio 2023.6.2.561. Significance was set at *p* < 0.05.

## Results

A total of 2,066 participants were included. The characteristics of the sample have been reported previously [1, 16]. Age-, gender- and habitation-adjusted 1-year prevalence estimates, also reported previously, were 67.9% for (any) headache, 26.3% for migraine, 34.1% for TTH, 3.0% for pMOH and 4.5% for other H15+ [1]. HY was reported by 12.0% [1].

### Symptom burden

Table 1 shows the symptom burden attributed to the various headache types.

Overall, mean headache frequency was 4.4 days/month with a mean duration of 22.6 h and a mean pTIS of 9.5%. Both headache frequency (*p* < 0.001) and duration (*p* = 0.02) were higher among females (4.9 days/month and 25.5 h) than males (2.9 days/month and 14.3 h). Mean headache intensity (overall 1.8, or moderate) was also higher among females (1.8) than males (1.6; *p* < 0.001; Table 1).

For pMOH and other H15+, mean headache frequency and pTIS were inevitably much higher (22.5 and 20.0 days/month and 61.8% and 41.9% respectively). Mean headache durations were reportedly also very high in these two groups (82.9 h and 126.0 h), indicating long-lasting rather than highly frequent attacks. Mean headache intensities were 2.5 for both pMOH and other H15+. There were no gender-related differences in symptom burden for pMOH or other H15+ (Table 1).

For migraine, mean headache frequency was 3.4 days/month, higher among females (3.6 days/month) than males (2.9 days/month; *p* = 0.04; Table 1). Headache duration (overall mean 18.4 h) did not differ significantly between genders. Mean pTIS (8.2% overall) was higher among females (8.7%) than males (6.0%; *p* = 0.01). Migraine-attributed lost health was therefore 2.7% (6.0%*0.441) for males and a greater 3.8% (8.7*0.441) for females (*p* = 0.01; Table 1). Mean headache intensity was 2.1 (males 2.0; females 2.1; *p* = 0.08; Table 1).

For TTH, mean headache frequency was 1.5 days/month, higher among females (1.7 days/month) than males (1.3 days/month; *p* = 0.001; Table 1). Mean headache duration was 6.8 h, also higher among females (7.7 h) than males (5.1 h; *p* = 0.003). Accordingly, so were mean pTIS (overall 1.7%; females 2.0%; males 1.0%; *p* < 0.001) and TTH-attributed lost health, although the latter was at the lower limit of estimation (females 0.1% [2.0*0.037]; males 0.0% [1.0%*0.037]; *p* < 0.001; Table 1). Mean headache intensity was 1.4 overall (mild), higher among females (1.4) than males (1.3; *p* = 0.01).

**Table 1:** Symptom burden, time in ictal state and headache-attributed lost health by headache type and gender

	Overall	Male	Female	Male vs. female
	mean±SEM, median			
**Frequency** (days/month)				
Any headache	4.4±0.2, 2.0	2.9±0.3, 0.6	4.9±0.2, 2.0	**F(1**,** 1446) = 25.4**, ***p*** <** 0.001**
pMOH	22.5±0.8, 20.0	23.0±2.0, 20.0	22.4±0.8, 20.0	F(1, 60) = 0.0, *p* = 0.83
Other H15+	20.0±0.8, 20.0	21.1±1.6, 20.0	19.7±0.9, 20.0	F(1, 93) = 0.4, *p* = 0.53
Migraine	3.4±0.1, 3.0	2.9±0.3, 2.0	3.6±0.2, 3.0	**F(1**,** 583) = 4.4**, ***p* = 0.04**
TTH	1.5±0.1, 0.6	1.3±0.1, 0.5	1.7±0.1, 0.6	**F(1**,** 703) = 7.3**, ***p*** **= 0.001**
**Duration** (hours)				
Any headache	22.6±2.2, 6.0	14.3±3.3, 4.0	25.5±2.7, 8.0	**F(1**,** 1446) = 5.3**, ***p* = 0.02**
pMOH	82.9±24.5, 24.0	11.6±5.1, 5.0	89.2±26.6, 24.0	F(1, 60) = 0.7, *p* = 0.39
Other H15+	126.0±25.4, 24.0	135.6±67.7, 8.0	123.9±27.5, 24.0	F(1, 93) = 0.0, *p* = 0.86
Migraine	18.4±0.7, 24.0	15.9±1.3, 10.0	19.0±0.8, 24.0	F(1, 583) = 3.0, *p* = 0.08
TTH	6.8±0.4, 2.0	5.1±0.5, 2.0	7.7±0.6, 3.0	**F(1**,** 703) = 9.0**, ***p*** **= 0.003**
**Intensity** (not bad-quite bad-very bad; equated to 1, 2, 3)				
Any headache	600-608-240 (mean = 1.8)	210-127-41 (mean = 1.6)	390-481-199 (mean = 1.8)	**X2(2**, ***N*** = 1448) = 43.3, ***p*** < **0.001**
pMOH	2-27-33 (mean = 2.5)	0-3-2 (mean = 2.4)	2-24-31 (mean = 2.5)	X2(2, *N* = 62) = 0.7, *p* = 0.71
Other H15+	13-47-35 (mean = 2.2)	3-9-5 (mean = 2.1)	10-38-30 (mean = 2.3)	X2(2, *N* = 95) = 0.6, *p* = 0.74
Migraine	110-325-150 (mean = 2.1)	26-54-31 (mean = 2.0)	84-271-119 (mean = 2.1)	X2(2, *N* = 585) = 3.0, *p* = 0.22
TTH	475-208-22 (mean = 1.4)	181-61-3 (mean = 1.3)	294-147-19 (mean = 1.4)	**X2(2**, ***N*** = 703) = 9.4, ***p* = 0.01**
**Proportion of time in ictal state** (%)				
Any headache	9.5±0.5, 1.4	4.9±0.8, 0.5	11.1±0.7, 1.6	**F(1**,** 1446) = 26.9**, ***p*** <** 0.001**
pMOH	61.8±4.3, 66.7	39.4±18.8, 13.9	63.8±4.4, 66.7	F(1, 60) = 2.4, *p* = 0.13
Other H15+	41.9±4.0, 20.8	44.6±10.6, 16.7	41.4±4.3, 20.8	F(1, 93) = 1.0, *p* = 0.76
Migraine	8.2±0.4, 3.8	6.0±0.8, 2.2	8.7±0.5, 4.6	**F(1**,** 583) = 6.6**, ***p* = 0.01**
TTH	1.7±0.1, 0.3	1.0±0.1, 0.2	2.0±0.2, 0.4	**F(1**,** 703) = 12.5**, ***p*** <** 0.001**
**Lost health** (%)(measured as pTIS x DW)				
Migraine	3.6±0.2, 1.7	2.7±0.3, 1.0	3.8±0.2, 2.0	**F(1**,** 583) = 6.6**, ***p* = 0.01**
TTH	0.1±0.0, 0.0	0.0±0.0, 0.0	0.1±0.0, 0.0	**F(1**,** 703) = 12.5**, ***p*** <** 0.001**

pTIS: proportion of time in ictal state; pMOH: probable medication-overuse headache; H15+: headache on ≥ 15 days/month; TTH: tension-type headache; DW: disability weight for the ictal state of the disorder [19]; significant p-values are emboldened

**Table 2:** Impaired participation (measured with HALT-30 index) by headache type and gender

	Overall	Male	Female	Male vs. female
	Lost days/month mean±SEM, median			
**HALT questions 1 and 2** (relating to paid work)				
Any headache	0.1±0.0, 0.0	0.3±0.1, 0.0	0.1±0.0, 0.0	**F(1**,** 1445) = 7.8**, ***p*** **= 0.005**
pMOH	0.9±0.5, 0.0	3.6±3.6, 0.0	0.7±0.4, 0.0	F(1, 60) = 2.7, *p* = 0.11
Other H15+	0.9±0.5, 0.0	3.5±2.7, 0.0	0.3±0.2, 0.0	**F(1**,** 93) = 6.5**, ***p* = 0.01**
Migraine	0.1±0.0, 0.0	0.3±0.1, 0.0	0.0±0.0, 0.0	**F(1**,** 583) = 17.0**, ***p*** < **0.001**
TTH	0.0±0.0, 0.0	0.0±0.0, 0.0	0.0±0.0, 0.0	F(1, 702) = 5.8, *p* = 0.20
	**F(3**,** 1442) = 13.5**, ***p*** <** 0.001**			
**HALT questions 3 and 4** (relating to household chores)				
Any headache	1.0±0.1, 0.0	0.4±0.2, 0.0	1.2±0.1, 0.0	**F(1**,** 1446) = 12.4**, ***p*** <** 0.001**
pMOH	7.3±1.1, 4.0	4.0±4.0, 0.0	7.6±1.2, 4.0	F(1, 60) = 0.7, *p* = 0.39
Other H15+	4.3±0.9, 0.0	4.5±3.0, 0.0	4.2±0.9, 0.0	F(1, 93) = 0.0, *p* = 0.93
Migraine	0.9±0.1, 0.0	0.5±0.1, 0.0	1.0±0.1, 0.0	**F(1**,** 583) = 4.4**, ***p* = 0.04**
TTH	0.1±0.0, 0.0	0.0±0.0, 0.0	0.1±0.0, 0.0	F(1, 703) = 3.2, *p* = 0.07
	**F(3**,** 1443) = 126.4**, ***p*** < **0.001**			
**HALT question 5** (relating to social or leisure activities)				
Any headache	0.0±0.0, 0.0	0.0±0.0, 0.0	0.0±0.0, 0.0	F(1, 1445) = 0.1, *p* = 0.7
pMOH	0.4±0.1, 0.0	1.0±1.0, 0.0	0.3±0.1, 0.0	F(1, 60) = 1.9, *p* = 0.17
Other H15+	0.0±0.0, 0.0	0.0±0.0, 0.0	0.0±0.0, 0.0	F(1, 93) = 0.4, *p* = 0.51
Migraine	0.0±0.0, 0.0	0.1±0.1, 0.0	0.0±0.0, 0.0	F(1, 583) = 3.3, *p* = 0.07
TTH	0.0±0.0, 0.0	0.0±0.0, 0.0	0.0±0.0, 0.0	F(1, 702) = 0.0, *p* = 0.97
	**F(3**,** 1442) = 30.5**, ***p*** <** 0.001**			

HALT: headache-attributed lost time; pMOH: probable medication-overuse headache; H15+: headache on ≥ 15 days/month; TTH: tension-type headache; significant p-values are emboldened

**Fig. 1:**
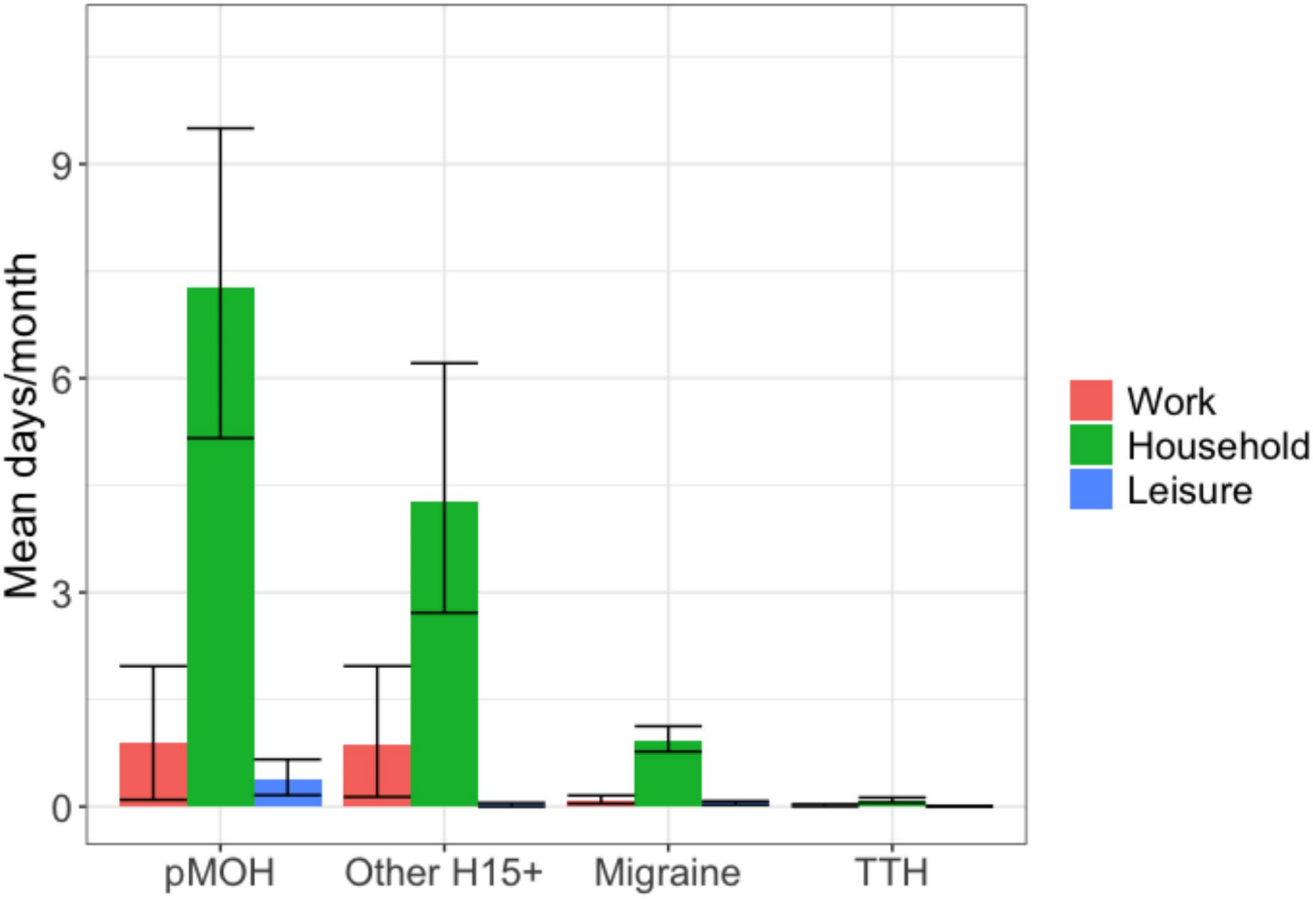
Impaired participation in paid (red) and household (green) work and social or leisure activities (blue) by headache type (error bars are 95% confidence intervals; pMOH: probable medication-overuse headache; H15+: headache on ≥ 15 days/month; TTH: tension-type headache)

### Impaired participation

Table 2 and Fig. 1 show headache-attributed impaired participation.

Overall, those with headache lost, on average, 0.1 days from paid work, 1.0 days from household work and 0.0 days from social or leisure activities in the preceding month (Table 2). Males lost more days from paid work than females (0.3 vs. 0.1 days; *p* = 0.005), whereas the opposite was true for household work (0.4 vs. 1.2; *p* < 0.001).

Those with pMOH lost, on average, 0.9 days from paid work, 7.3 days from household work and 0.4 days from social or leisure activities, with no significant gender-related differences (Table 2). Those with other H15 + lost on average 0.9 days from paid work (males [3.5] more than females [0.3]; *p* = 0.01), 4.3 days from household work and 0.0 days from social or leisure activities.

Those with migraine lost, on average, 0.1 days from paid work, 0.9 days from household work and 0.0 days from social or leisure activities (Table 2). Males lost more days from paid work than females (0.3 vs. 0.1; *p* < 0.001), whereas the opposite was true for household work (0.5 vs. 1.0; *p* = 0.04). Those with TTH lost very little time: on average, 0.0 days from paid work, 0.1 days from household work and 0.0 days from social or leisure activities (Table 2), with no significant gender-related differences.

Headache type was a significant predictor for impaired participation in all domains (Table 2; Fig. 1). For all headache types, losses from household work were always significantly and substantially greater than those from paid work or from social or leisure activities (Fig. 1).

### Headache yesterday

Table 3 shows symptom burden and impaired participation attributed to HY. Reported mean duration of HY was 15.6 h, similar among males (14.9 h) and females (15.7 h; *p* = 0.79). Median values (overall 6.0 h) indicated skewed data. Headache intensity was also similar among males (mean: 1.8) and females (1.7; *p* = 0.74).

Of the 247 participants reporting HY, 176 (71.3%) could do everything of their normal activities, 29 (11.7%) more than half, 28 (11.3%) less than half and 14 (5.7%) nothing. Analyzed in the same manner as HALT data (dichotomized as 0 or 100%), overall impaired participation with HY was estimated to be 17%.

**Table 3:** Symptom burden and impaired participation from headache yesterday (*n* = 247)

	Overall	Male	Female	Male vs. female
**Duration** (hours) mean±SEM, median	15.6±1.1, 6.0	14.9±2.7, 5.0	15.7±1.2, 6.0	F(1, 245) = 0.1, *p* = 0.79
**Intensity** ^1^				
not bad (n) quite bad (n) very bad (n) mean^2^	102 101 42 1.8	15 19 7 1.8	87 82 35 1.7	*X*^*2*^(2, *N* = 245) = 0.6, *p* = 0.74
**What done**				
everything (n) more than half (n) less than half (n) nothing (n)	176 29 28 14	34 2 4 2	142 27 24 12	*X*^*2*^(3, 247) = 3.0, *p* = 0.39

^1^ data missing from two participants; ^2^ equating to 1, 2, 3, and treating as though continuous data

### Overall burden

**Fig. 2:**
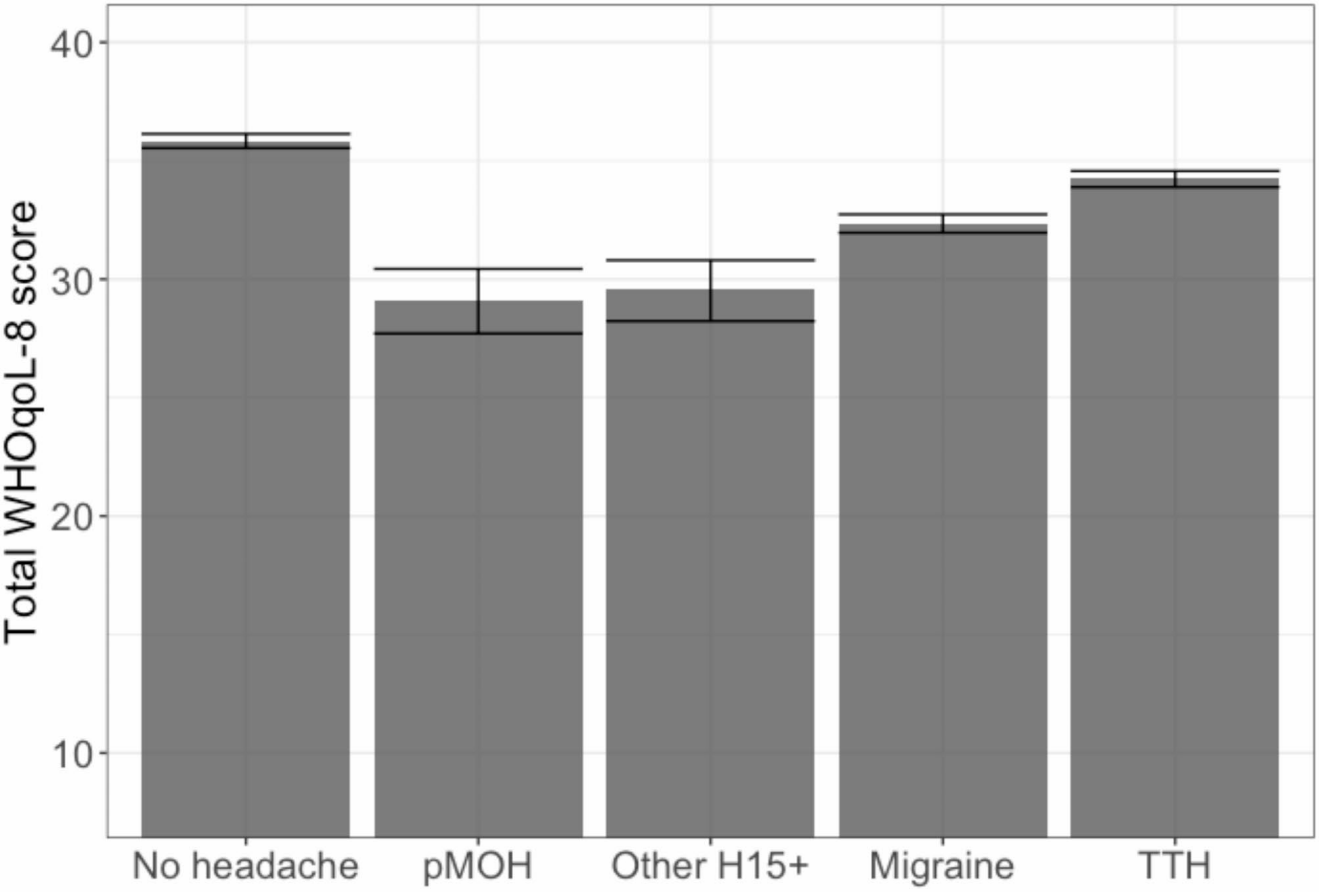
Mean reported quality of life (WHOQoL-8, scale 8–40) by headache type (error bars: 95% CI; pMOH: probable medication-overuse headache; H15+: headache on ≥ 15 days/month; TTH: tension-type headache)

Mean QoL was significantly higher among those with no headache (35.8/40) than among those with any of the headache types (Table 4; Fig. 2). Those with pMOH (29.1) or other H15+ (29.6) reported significantly lower mean QoL than those with migraine (32.4), who reported significantly lower QoL than those with TTH (34.2; Table 4; Fig. 2).

**Table 4:** Quality of life (measured with WHO QoL-8) and willingness to pay by headache type

Headache type	Quality of life (scale 8–40) mean±SEM, median	Willingness to pay (INR/month) mean±SEM, median
No headache	35.8±0.2, 37.0	-
pMOH	29.1±0.7, 30.0	690.5±104.6, 500.0
Other15+	29.6±0.7, 30.0	1,221.3±253.9, 300.0
Migraine	32.4±0.2, 33.0	730.4±212.4, 100.0
TTH	34.2±0.2, 35.0	258.4±83.6, 0.0
	**F(4**,** 2060) = 82.5**, ***p*** <** 0.001**	**F(3**,** 1435) = 3.0**, ***p* = 0.03**

pMOH: probable medication-overuse headache; H15+: headache on ≥ 15 days/month; TTH: tension-type headache; significant p-values are emboldened; at the time of the study, USD 1 = INR 65.58 [22]

**Fig. 3:**
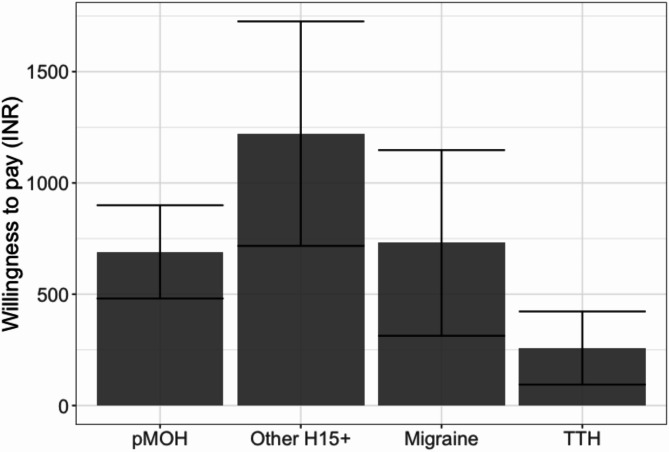
Mean willingness to pay for treatment (INR/month) by headache status (error bars: 95% CI; pMOH: probable medication-overuse headache; H15+: headache on ≥ 15 days/month; TTH: tension-type headache; at the time of the study, USD 1 = INR 65.58 [22])

Headache type influenced WTP (Table 4; Fig. 3). More specifically, those with pMOH (INR 690.50/month) or other H15+ (INR 1,221.30/month) were willing to pay significantly more for headache treatment than those with TTH (INR 258.40/month) although WTP among those with migraine (INR 730.40/month) was not statistically different from WTP among those with any of the other headache types. It should be noted that CIs were wide (Fig. 3), the data skewed (most medians considerably lower than means) and all values were the equivalent of < USD 20 [22].

### Health care needs assessment

Of the 2,066 participants, 622 (30.1%) fulfilled one or more of our criteria for likelihood of benefit from professional health care (Table 5), our definition of “need”. Corrected for age and gender, the estimated proportion was 26.0% of all adults aged 18–65 years. For H15+, migraine and TTH, proportions were 6.4%, 16.1% and 3.5% respectively.

### Population-level estimates

Table 6 shows population-level estimates of pTIS and impaired participation. From 1-year prevalence and recalled headache frequency and usual headache duration, we estimated that 5.5% of all time among the population aged 18–65 years was spent with headache. Of this, migraine accounted for the largest part (2.0%), followed by other H15+ (1.7%), pMOH (1.4%) and lastly TTH (0.5%). The estimate based on HY was slightly higher (6.6% of all time spent with headache).

**Table 5:** Health-care needs assessment

Criterion fulfilled		Proportion of sample		Estimated proportion of adult population*
		*n*	%	% [95% CI]
1	Headache on ≥ 15 days/month	157	7.6	6.4 [5.4–7.6]
2	Migraine on ≥ 3 days/month	302	14.6	12.7 [11.3–14.2]
3	Migraine and pTIS > 3.3% and moderate-severe intensity	274^1^	13.3	11.3 [10.0-12.7]
4	Migraine and lost work and/or household days/3 months ≥ 3	177^2^	8.6	7.2 [6.1–8.4]
5	TTH and pTIS > 3.3% and moderate-severe intensity	60	2.9	2.4 [1.8–3.2]
6	TTH and lost work and/or household days/3 months ≥ 3	35^3^	1.7	1.5 [1.0-2.1]
One or more of criteria 1–6		622	30.1	26.0 [24.1–28.0]

*Age- and gender-corrected; ^1^of whom 231 also fulfilled criterion 2; ^2^of whom 124 also fulfilled criterion 2, 121 also fulfilled criterion 3 and 104 also fulfilled criteria 2 and 3; ^3^of whom 11 also fulfilled criterion 5

From HALT data, we estimated that, per person per month, 0.1 days were lost from paid work because of headache, 0.6 days from household work (pMOH, other H15 + and migraine each accounting for 0.2 days) and 0.0 days from social or leisure activities. From HY data, we estimated that 1.8% of all activity was lost to headache.

## Discussion

Having shown headache to be prevalent among adults aged 18–65 years in Delhi and NCR of India [1], here we report high levels of burden attributed to it. People with headache in the past year spent, on average, 9.5% of their time with headache of moderate intensity (1.8 on the scale of 1–3). Factoring in 1-year prevalence and correcting for age and gender, we estimated that 5.5% of all time in this population of northern India was spent with headache of some sort, mostly H15+ (3.1%; pMOH 1.4%; other H15 + 1.7%), followed by migraine (2.0%). People with pMOH also reported more intense headache (mean 2.5, on a 1–3 scale) than those with migraine (2.1) or TTH (1.4). Very probably as a consequence of greater pTIS with more intense headache, H15 + was associated with higher levels of impaired participation and lower QoL than migraine or TTH. In our previous study in Karnataka State, in southern India [4], we also found a substantially higher individual burden among those with H15 + than among those with migraine or TTH.

**Table 6:** Proportion of time in ictal state and impaired participation at population level by headache type and timeframe of enquiry (adjusted for age and gender)

Headache type	Estimated pTIS (%)		Estimated impaired participation			
	According to 1-year prevalence and reported average frequency and usual duration	According to prevalence and duration of headache yesterday	According to HALT data (lost days/month)			According to headache yesterday
			Paid work	Household work	Social or leisure activities	Lost activity (%)
Any headache	5.5	6.6	0.1	0.6	0.0	1.8
pMOH	1.4		0.0	0.2	0.0	
Other H15+	1.7		0.1	0.2	0.0	
Migraine	2.0		0.0	0.2	0.0	
TTH	0.5		0.0	0.0	0.0	

pTIS: proportion of time in ictal state; HALT: headache-attributed lost time; pMOH: probable medication-overuse headache; H15+: headache on ≥ 15 days/month; TTH: tension type headache

It was unsurprising that migraine was more burdensome than TTH, although the large difference in pTIS (8.2% for migraine, 1.7% for TTH) was not predictable. Despite its high prevalence of 34.1%, TTH therefore contributed little to overall population-level pTIS (0.5%). The contribution from migraine was four times as high (2.0%).

Females with migraine or TTH spent significantly more time with headache (8.7% and 2.0% respectively) than males (6.0% and 1.0%). For TTH this was driven by both higher headache frequency and longer headache duration; for migraine, only frequency was significantly different between the genders.

Factoring in DWs provided by GBD [19], we found that migraine accounted for very substantial health loss (3.6%) at individual level, whereas, for TTH, health loss was at the lower limit of estimation (0.1%). Both measures of overall burden (QoL and WTP) reflected this, each indicating greatest individual burden among those with H15+, followed by migraine and TTH least. Despite that both measures are highly subjective, and QoL has no meaningful units, we believe they provide a broad insight into overall disease burden that is not gained from other measures.

For all headache types, participation was more impaired in household work than in paid work. This is in line with previous studies [10–13], and is plausibly explained by household chores being more optional (or more readily postponed) than income-generating work. The losses from paid work (males: 0.3 days/month; females 0.1 days/month) might be considered very small, certainly lower than we observed in Karnataka State [4]. Losses from paid work are influenced by nature and perceived importance of the work, and by fears of lost income (which may be offset by sick pay) and of employment insecurity. All may be subject to cultural, industrial, economic and political differences between the two regions. Gender differences in employment were probably the reason why males lost more days from paid work than females, and vice versa household work.

Impaired participation in social or leisure activities was close to unmeasurably low. In Benin, where we found similar, we offered four possible explanations [12]: that little time was given to social or leisure activities; that headache was not allowed to disrupt these activities; that losses from them were not well remembered; that there was disinclination to report such losses. Neither in Benin nor here can we know the true explanation.

Since this was a cross-sectional study with enquiry dependent upon recall, we were cognisant of likely recall error. We therefore included questions on HY, to which responses were expected to be free from recall error. Interestingly, our population-level estimate of pTIS from HY data (6.6%) was similar to, and a corroboration of, the estimate based on recall (headache frequency and usual duration: 5.5%). We are therefore reasonably confident that the proportion of all time spent with headache in this region of India was in this range 5.5–6.6%. For impaired participation, we used the 1-month version of HALT (HALT-30 [20]) rather than the 3-month version (HALT-90 [20]) used in Karnataka [2, 4], mitigating recall error here to the extent that this increased with recall period. However, estimates of impaired participation from HALT and HY (1.8% across all domains of activity) cannot be directly compared: denominators (days of activity that might be lost) are uncertain for the former (possibly but not necessarily 20–22/month for paid work, but unknown for household work and for social or leisure activities); this was not so for HY, which related to whatever had been planned on one specific day (but not in each separate domain).

A main aim of this study was to assess need for headache-related health care, informing local and national makers of health and economic policies. One quarter (26%) of the population aged 18–65 years would be expected to benefit from effective health care (such as might be provided through structured headache services [23, 24]), meeting our criteria for need. Most had migraine (16.1%, with 12.7% meeting usual criteria for requiring preventative medication), but the 6.4% with H15 + also had undeniable need for professional health care. The latter included pMOH, a preventable disorder, but prevention requires education. We presume these needs – for effective health care and education – to be unmet. A review is required of actual service provision in the region against the advocated standard [23]. This will inform proposals for change that should prove highly cost-effective in terms of expenditure per healthy life-year gained [24].

### Strengths and limitations

We have previously reported the major strengths and limitations of this study [1, 16]. Strengths, in summary, were the use of established methodology [14, 15], a validated translation of the HARDSHIP questionnaire [16], and a large and representative sample of the general population. Pre-pilot and pilot studies were performed [16]. Quality-control measures were in place [16].

Among limitations were the participating proportion of 68.0% [16], relatively low, and lower in urban areas (52.9%), but this was far from invalidating. The reasons for this and its implications have already been discussed [1, 16]. Recall error is always likely in studies dependent on recall, but we mitigated this by use of HALT-30 rather than HALT-90 [20], and by including questions on HY. Since only one diagnosis was allowed in each participant (for the most bothersome headache when more than one type was reported), the burden attributed to TTH may have been underestimated.

## Conclusions

Headache disorders in India are not only prevalent but also associated with high burden, with 5.5–6.6% of all time spent with headache. pMOH, an avoidable illness, accounts for one quarter of this time. An estimated one quarter of the adult population of this region of India are in need of professional health care, which would best be met by putting structured headache services in place, with their basis in primary care [23, 24].

## Data Availability

The original data are held on file at the All India Institute of Medical Sciences, New Delhi, India, and the analytical subset at Norwegian University of Science and Technology, Trondheim, Norway. Once analysis and publications are completed, they will be freely available for non-commercial purposes to any person requesting access in accordance with the general policy of the Global Campaign against Headache.

## Abbreviations

CI: confidence interval
d/m: days/month
GBD: Global Burden of Disease (study)
HALT: Headache-Attributed Lost Time (index)
HARDSHIP: Headache-Attributed Restriction, Disability, Social Handicap and Impaired Participation (questionnaire)
HY: headache yesterday
ICHD: International Classification of Headache Disorders
INR: Indian rupee
LTB: *Lifting The Burden*
MOH: medication-overuse headache
pMOH: probable MOH
pTIS: proportion of time in ictal state
QoL: quality of life
SEAR: South East Asia Region
TTH: tension-type headache
USD: United States dollar
WHOQoL-8: World Health Organization 8-item quality of life (questionnaire)
WTP: willingness to pay
